# Can classification strategies improve automated cervical vertebral maturation staging? A comparative study

**DOI:** 10.1038/s41598-026-46004-z

**Published:** 2026-03-30

**Authors:** Soo-Young Lee, Jiho Ryu, Ye-Hyun Kim, You-Sun Lee, Seok-Ki Jung

**Affiliations:** 1 Department of Orthodontics, Korea University Graduate School of Medicine, Seoul, Republic of Korea; 2 Department of Orthodontics, School of Dentistry, Dental Research Institute, Seoul National University, Seoul, Republic of Korea; 3 Department of Orthodontics, Korea University Anam Hospital, 73 Goryeodae-ro, Seongbuk-gu, Seoul, 02841 Republic of Korea; 4 Department of Orthodontics, Korea University Guro Hospital, 148 Gurodong-ro, Guro-gu, Seoul, 08308 Republic of Korea

**Keywords:** Artificial intelligence, Cervical vertebral maturation, Deep learning, Orthodontic diagnosis, Skeletal maturity, Computational biology and bioinformatics, Health care, Mathematics and computing, Medical research

## Abstract

Accurate assessment of skeletal maturity is essential in determining orthodontic treatment timing. Cervical vertebral maturation (CVM) staging is commonly used, but inter-observer variability remains a major obstacle. Recently, artificial intelligence (AI) has been used to provide more consistent and faster radiographic assessments. This study aimed to compare various deep learning approaches and investigate how different training strategies impact model performance in automated CVM staging. A total of 1,750 lateral cephalometric radiographs were independently evaluated and labeled by two board-certified orthodontists, with discrepancies resolved by consensus. This dataset was then divided into a training set (*n* = 1,600; stratified 5-fold cross-validation) and a held-out test set (*n* = 150). First, we compared the end-to-end 6-stage model (LS6) with landmark-guided 6-stage models (LM6_1 and LM6_2, collectively referred to as LM6) to test the effect of structural priors. Then, we evaluated a fine-to-coarse 3-stage model (LS6_3), trained on 6 stages and then aggregated for 3-stage classification, against a direct 3-stage model (LS3) to assess the impact of label granularity. For 6-stage classification, LS6 achieved 67.3% accuracy (κ = 0.912), outperforming LM6_1 (58.8%) and LM6_2 (64.4%). Tolerance-based analysis demonstrated high ± 1-stage accuracy for all models, with LS6 achieving 94.3% and LM6 models achieving 92.9% (LM6_1) and 92.8% (LM6_2), confirming that misclassifications were predominantly between adjacent stages. For 3-stage classification, LS6_3 (79.3%) showed slightly higher accuracy than LS3 (78.8%). In the Grad-CAM analysis, LS6_3 showed a more concentrated focus on key vertebral features compared to LS3. These findings suggest that landmark-based priors may not necessarily enhance CVM staging performance, and that fine-grained training encourages more anatomically focused feature learning. These results provide preliminary evidence for optimizing AI training protocols in skeletal maturity assessment.

## Introduction

In clinical orthodontics and dentofacial orthopedics, achieving functional occlusion and long-term stability largely depends on the precise timing of intervention^1^. For pediatric and adolescent patients, the pubertal growth spurt provides a clinically important window to correct skeletal discrepancies and modulate growth patterns. Skeletal growth is non-linear and displays individual variation; thus, chronological age is not a reliable predictor of biological maturity. Consequently, clinicians must rely on skeletal maturation indicators to pinpoint an individual’s maturity level, which directly dictates appliance selection and treatment planning^2^.

While hand-wrist radiography has long been the gold standard for assessing skeletal age^3^, it requires a dedicated radiograph separate from the routine orthodontic diagnostic series. To avoid this additional radiation exposure, the Cervical Vertebral Maturation (CVM) method has emerged as a practical alternative, as it utilizes the lateral cephalometric radiographs that are already obtained during standard orthodontic diagnosis^4^. Despite its high correlation with hand-wrist maturation^5,6^, the 6-stage CVM classification is frequently criticized for its considerable inter-observer variability^7^. The subtle morphological shifts between stages—particularly during the peak growth period—often lead to inconsistent diagnostic outcomes, which poses a challenge for clinical standardization^8,9^.

To enhance diagnostic objectivity, deep learning algorithms, particularly Deep Convolutional Neural Networks (DCNNs), have been increasingly explored in orthodontics, with their clinical utility demonstrated in areas such as extraction decision-making and orthognathic surgery planning^10–12^. More recently, these techniques have been applied to automated CVM staging^13^. However, despite reported improvements in accuracy, clinical implementation is hindered by a lack of evidence-based model training protocols.

Two key questions remain unresolved. First, it is unclear whether integrating structural priors—predefined knowledge provided to the model as guidance during training—helps the model learn morphological features, or instead causes negative transfer, where prior knowledge degrades rather than improves performance. Second, the influence of label granularity—the number of categories used for classification—has not been rigorously examined. Clinically, a simplified 3-stage classification (pre-peak, peak, and post-peak) is often sufficient for treatment timing, yet training on the full 6-stage scale may help the model capture finer morphological transitions near stage boundaries. Whether this finer-grained training confers a meaningful advantage remains an open question^14,15^. Through a systematic ablation analysis of training protocols, this study aims to provide empirical evidence on how different AI configurations influence diagnostic performance and model behavior. The clinical relevance of this work lies in optimizing these AI training strategies to mitigate the diagnostic inconsistency of manual CVM staging. Such comparative evidence is a necessary step toward optimizing deep learning models that can eventually assist clinicians in identifying the pubertal growth peak more consistently, potentially guiding the timing for growth modulation therapies such as functional appliance treatment in Class II malocclusion^16,17^.

Based on these considerations, we established the following null hypothesis: that neither the integration of landmark-based structural priors nor the granularity of maturation labels (6-stage vs. 3-stage) would lead to significant differences in the classification performance or the anatomical interpretability of the deep learning models. To test this hypothesis, this study evaluates how different learning approaches and prior knowledge influence the performance and interpretability of automated CVM staging models. Specifically, we compare autonomous feature learning against landmark-guided transfer learning and assess how label granularity modulates anatomical attention patterns.

## Method

### Study design and ethical considerations

This study was designed as a retrospective investigation and was conducted in accordance with the principles of the Declaration of Helsinki. The study protocol was reviewed and approved by the Institutional Review Board (IRB) of Korea University Guro Hospital (IRB No. 2025-GR-0022). Due to the retrospective nature of the study and the use of de-identified radiographic data, the requirement for informed consent was waived by the IRB.

### Dataset and preprocessing

This study included a total of 1,750 lateral cephalometric radiographs from patients who underwent orthodontic diagnosis at the Department of Orthodontics, Korea University Guro Hospital between 2014 and 2024. The exclusion criteria were as follows: (1) radiographs of significantly poor quality due to blurring or motion artifacts, and (2) patients with diagnosed craniofacial anomalies or syndromes. The mean age of the included subjects was 12.9 ± 4.1 years. To ensure data privacy, all images were anonymized and assigned unique identification numbers prior to analysis.

**Fig. 1:**
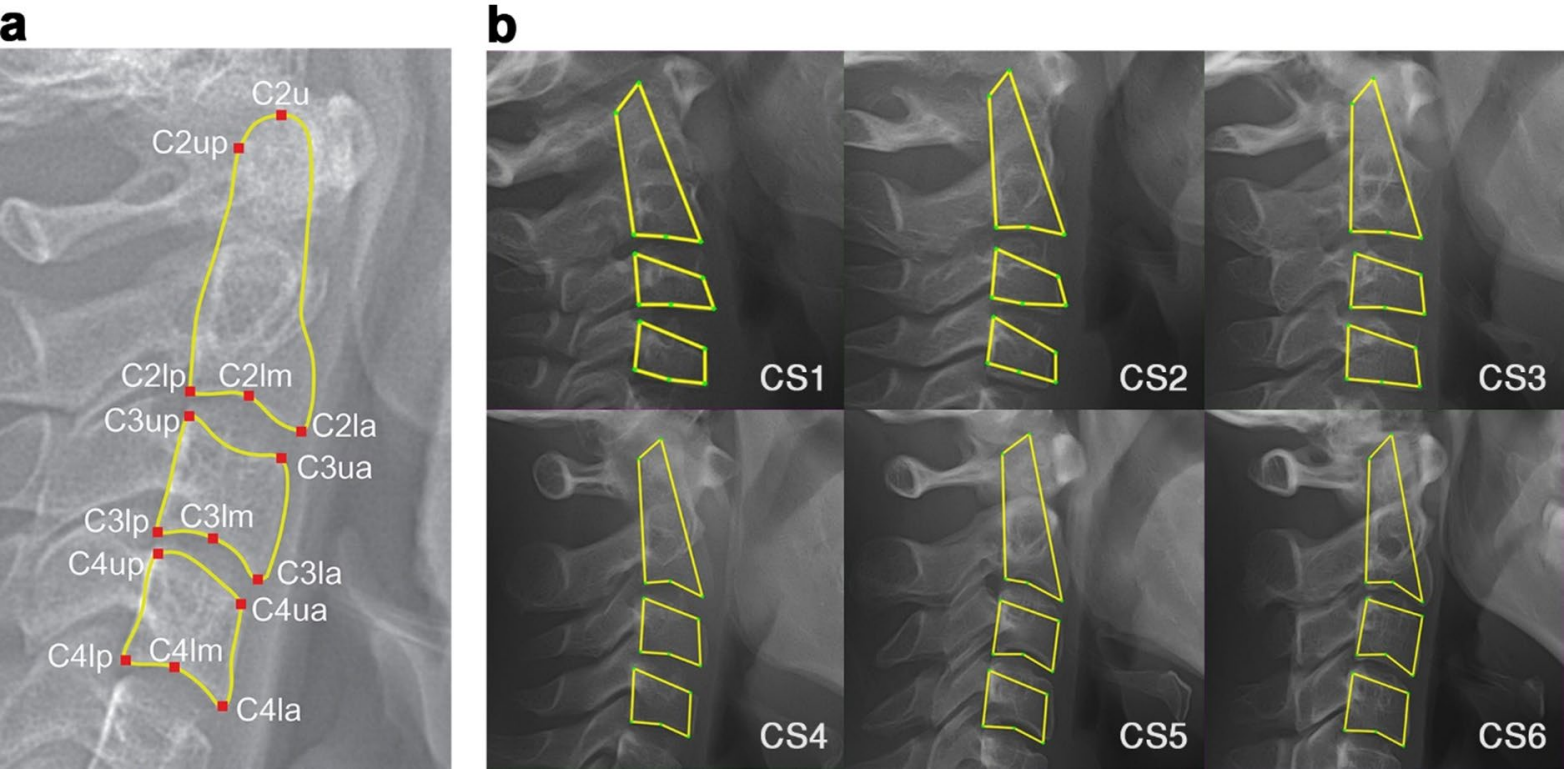
Anatomical landmarks and AI-driven Cervical Vertebral Maturation (CVM) staging. (**a**) Fifteen anatomical landmarks identified on the second to fourth cervical vertebrae (C2, C3, and C4). C2u, C2up, C2la, C2lm, C2lp on C2; C3ua, C3up, C3la, C3lm, C3lp on C3; C4ua, C4up, C4la, C4lm, C4lp on C4; u=upper, l=lower, a=anterior, m=middle, p=posterior. (**b**) Representative results of AI-based CVM classification across Cervical Stages (CS) 1 to 6, illustrating the morphological progression from trapezoidal to rectangular vertebral body configurations consistent with the Baccetti criteria. Images are arranged in order from left to right and top to bottom, corresponding to CS1 through CS6.

The reference standard for Cervical Vertebral Maturation (CVM) staging and anatomical landmark detection was established through independent assessment by two board-certified orthodontists. For each radiograph, the clinicians determined the Cervical Stage (CS1–CS6) and identified 15 anatomical landmarks on the C2–C4 vertebrae (Fig. 1a). CVM staging followed the morphological criteria defined by Baccetti et al.^17^, assessing the inferior borders of the vertebral bodies for concavity and the shapes of C3 and C4, which evolve from trapezoidal to vertical rectangular configurations (Fig. 1b). Any diagnostic discrepancies between the two specialists were resolved through collaborative consensus to establish a definitive ground-truth classification. Prior to consensus resolution, the inter-observer agreement between the two orthodontists showed substantial agreement (weighted Cohen’s κ = 0.81).

The dataset comprised CS1 (*n* = 261), CS2 (*n* = 215), CS3 (*n* = 227), CS4 (*n* = 346), CS5 (*n* = 334), and CS6 (*n* = 367). For model evaluation, a test set of 150 images was randomly selected, maintaining the original proportionality of the stage distribution (Table 1). The remaining 1,600 images were utilized as the training set, to which a stratified 5-fold cross-validation protocol was applied^18^. Within each fold, data were further split into training and validation sets at an 80:20 ratio (*n* = 1,280 and 320, respectively). To prevent overfitting and ensure model stability, data augmentation techniques were applied using affine transformations, random window cropping, gamma correction, and adjustments to brightness and contrast.

**Table 1:** Distribution of CVM stages across datasets.

CVM stage	Learning set	Test set	Total
CS1	239	22	261
CS2	197	18	215
CS3	207	20	227
CS4	316	30	346
CS5	305	29	334
CS6	336	31	367
Total	1,600	150	1,750

(CVM, cervical vertebral maturation; CS, cervical stage)

### ROI detection

**Fig. 2:**
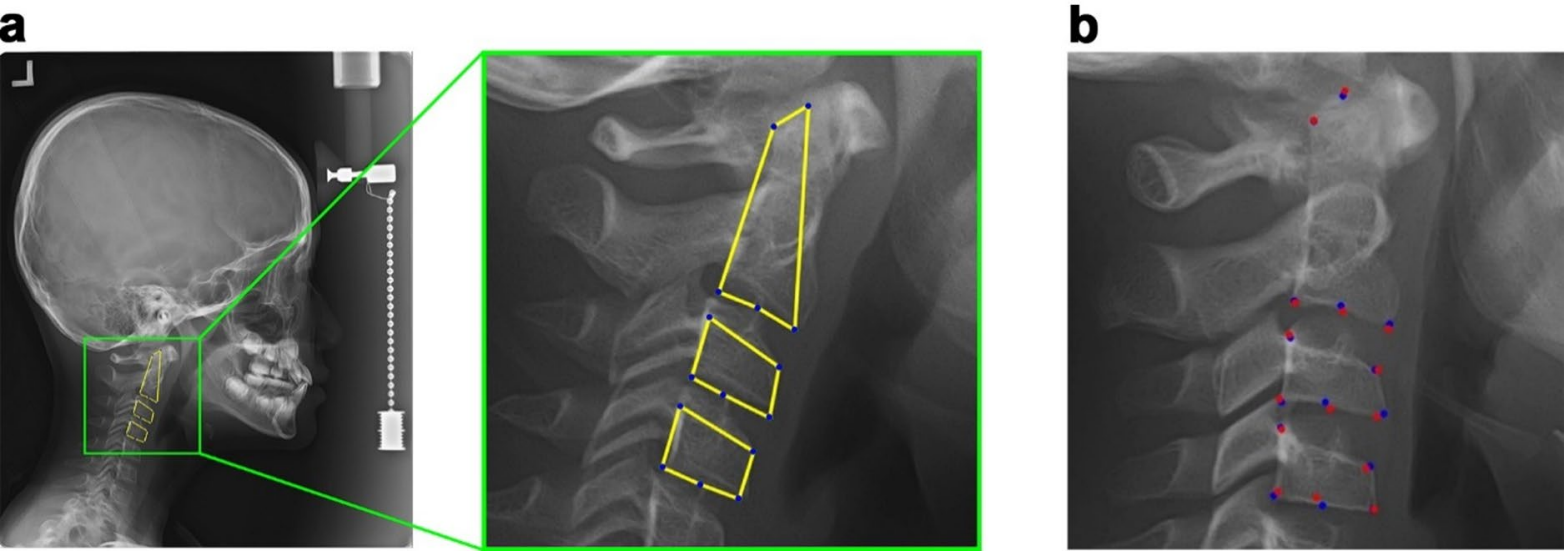
Automated region of interest (ROI) detection and landmark detection on lateral cephalometric radiograph. (**a**) Original lateral cephalometric radiograph with the detected C2–C4 ROI indicated by a yellow-green bounding box. The isolated and resized (224 × 224 pixels) ROI is shown alongside. The square format preserves vertebral aspect ratios critical for accurate CVM classification. (**b**) Coordinates for the 15 anatomical points on C2-C4 vertebrae are shown in red (ground-truth) and blue (predicted).

A Faster Region-based Convolutional Neural Network (Faster R-CNN) model was employed to detect the region of interest (ROI) encompassing the C2–C4 cervical vertebrae on each lateral cephalometric radiograph (Fig. 2a). The ROI was configured as a square to preserve the height-to-width ratio of the vertebral bodies, which constitutes a primary morphological indicator in CVM staging. To increase the spatial resolution devoted to vertebral structures, the bounding box was tightly fitted to the C2–C4 region. The detected ROI was then cropped and resized to 224 × 224 pixels to match the input dimensions of the pretrained DCNN backbone^19^.

### Classification models and training strategies

This study examines two independent factors in automated CVM staging: (1) the effect of structural priors, comparing end-to-end learning against landmark-guided transfer learning for 6-stage classification, and (2) the influence of label granularity, comparing direct 3-stage training against 3-stage results derived from a 6-stage model. To investigate these factors, three training strategies were designed. All models were initialized with weights pre-trained on the ImageNet database to provide a uniform baseline of visual features.

#### LS6: end-to-end learning for 6-stage classification

**Fig. 3:**
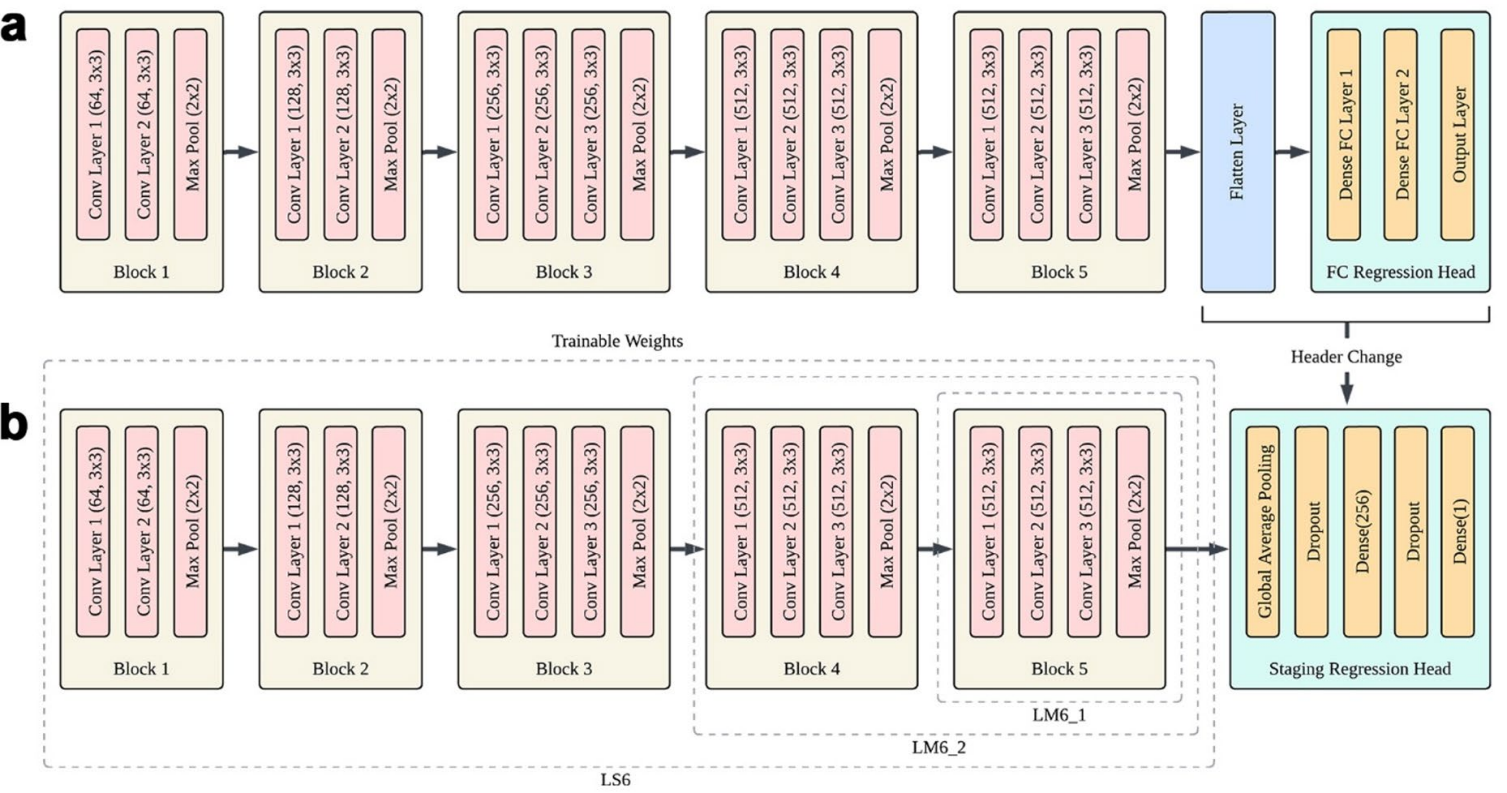
Architectures and training strategies of the DCNN models. (**a**) Landmark Detection model (**b**) CVM Staging Classification Model. The regression head predicting landmark coordinates is replaced with a Global Average Pooling layer, dropout layer, and dense layer with scalar output for CVM stage classification. LS6 unfroze all blocks down to Block 1 for full parameter updates, LM6_1 unfroze up to Block 5, and LM6_2 further unfroze to Block 4 with reduced learning rate to leverage landmark-based morphological features.

The final layers of the ImageNet-pretrained DCNN backbone were replaced with a staging head designed for CVM classification. The staging head consisted of a Global Average Pooling (GAP) layer to aggregate spatial feature maps into a global representation, a dropout layer to prevent overfitting, and a final dense layer with a single scalar output for ordinal stage prediction (Fig. 3b). All architectural blocks down to Block 1 were unfrozen for full-parameter updates.

#### LM6: landmark-guided learning for 6-stage classification

To leverage morphological features as anatomical priors, the final layers of the ImageNet-pretrained DCNN backbone were replaced with a landmark regression head (Fig. 3a). This head consisted of a flatten layer, a dense layer, and an output layer with 30 units corresponding to the x and y coordinates of 15 anatomical landmarks on C2–C4 (Fig. 1a), yielding predicted coordinates for the 15 landmarks (Fig. 2b). All coordinate values were normalized between 0 and 1, and the model was optimized using a Mean Squared Error (MSE) loss function. Subsequently, the landmark regression head was replaced with the same staging head used in LS6. In LM6_1, layers from the output head back to Block 5 were unfrozen for preliminary fine-tuning, while the earlier blocks (1–4) remained frozen to preserve the pre-trained landmark priors (Fig. 3b). In LM6_2, a more extensive refinement was conducted by further unfreezing layers back to Block 4 with a reduced learning rate to promote stable convergence and prevent disruption of pre-trained feature representations^20^.

#### LS3: end-to-end learning for 3-stage classification

LS3 utilized the same full-parameter, end-to-end protocol as LS6 but was trained for a simplified 3-stage classification. The six CVM stages were restructured into three clinically significant categories: Pre-pubertal (CS1–CS2), Pubertal (CS3–CS4), and Post-pubertal (CS5–CS6). The performance of LS3 was compared with LS6_3, which represents the LS6 outputs mapped into the same three categories, to assess the effect of label granularity.

### Optimization and training environment

The models were configured to perform scalar ordinal regression, outputting a normalized value between 0 and 1. To ensure stable convergence and account for morphological ambiguity in transitional stages, the Huber loss function was employed instead of MSE^21^.

Training was conducted on a Windows 10 workstation equipped with an NVIDIA RTX A6000 GPU (48 GB VRAM) and 256 GB RAM, using Keras 2.3.1 (Python 3.7.4) with a TensorFlow-GPU 2.5.0 backend^22^. Optimization was performed via the Adam optimizer with a batch size of 32 for up to 150 epochs. The initial learning rate was set to 1 × 10^− 3^, which was adjusted by a factor of 0.2 if the validation loss failed to improve by more than 1 × 10^− 6^ over three consecutive epochs. Training was terminated when the improvement in validation loss did not exceed 1 × 10^− 6^ for 8 epochs, and the model weights with the maximum validation accuracy were saved.

### Evaluation metrics

The quantitative performance of the models was measured using overall accuracy and tolerance-based accuracy (within ± 1 and ± 2 stages). To account for the ordinal nature of skeletal maturation, quadratic weighted Cohen’s kappa was calculated. The quadratic weighting scheme was selected as the primary metric, as it assigns greater penalty to larger classification errors, which is appropriate for ordinal staging. The 95% confidence intervals for accuracy and quadratic weighted kappa was computed from the 5-fold cross-validation results using the t-distribution (df = 4). Sensitivity, specificity, precision, F1-score, and the area under the receiver operating characteristic curve (AUC) were reported for each stage.

To assess the anatomical validity of each model’s decision-making process, Gradient-weighted Class Activation Mapping (Grad-CAM) was employed^23^. Grad-CAM heatmaps were generated for the test set, and the consistency of activation areas was evaluated relative to the key diagnostic features defined in the CVM method: (1) the inferior cortical borders of the C2–C4 vertebrae, (2) the concavities of the vertebral bodies, and (3) the morphological proportions associated with maturation-related shape changes.

## Results

### Landmark regression performance

The landmark detection model used for pre-training in the LM6 models achieved a mean radial error (MRE) of 1.284 ± 0.444 mm on the test set.

#### 6-Stage classification: end-to-end learning vs. landmark-guided learning (LS6 vs. LM6)

**Fig. 4:**
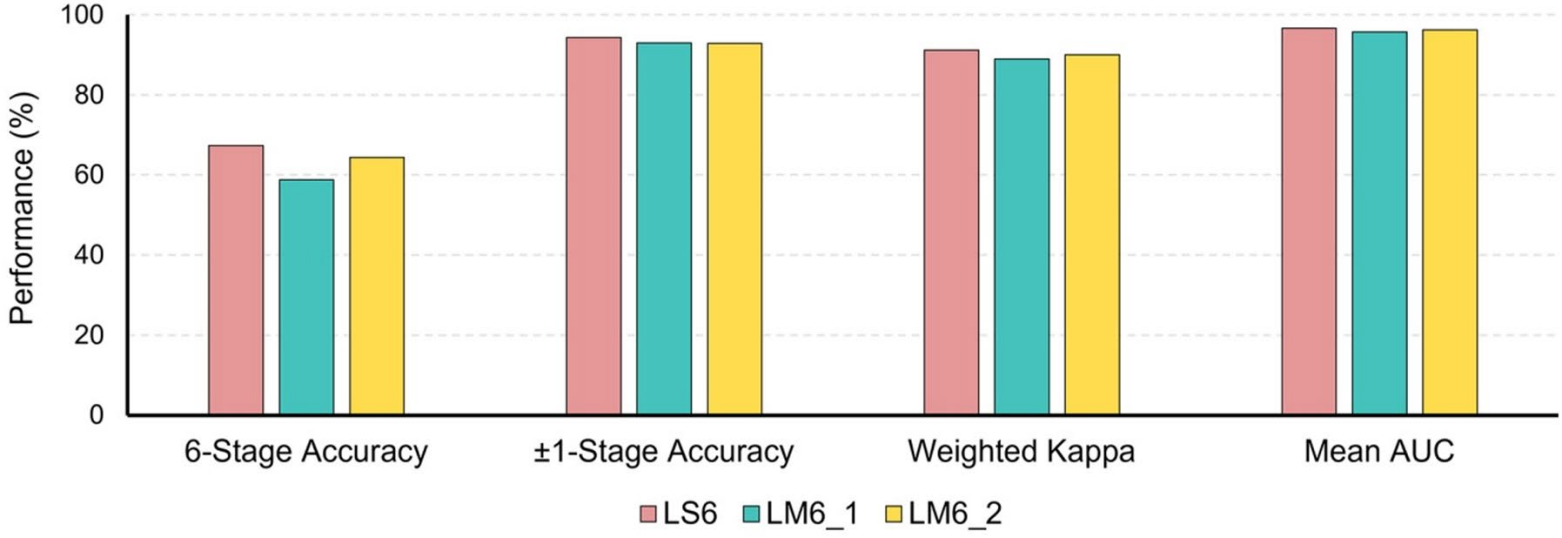
Performance comparison of 6-stage CVM classification models. Comparison of Accuracy, ± 1-stage Accuracy, Quadratic Weighted Kappa, and Mean Area Under the Curve (AUC) for LS6, LM6_1, and LM6_2. LS6 utilizes an autonomous image-based learning strategy, while LM6_1 and LM6_2 are landmark-integrated models. LS6 achieved 67.3% Accuracy, 94.3% ±1-stage Accuracy, 0.912 Weighted Kappa, and 0.966 Mean AUC, outperforming both LM6_1 (58.8%, 92.9%, 0.889, 0.957) and LM6_2 (64.4%, 92.8%, 0.900, 0.962) across all metrics.

LS6 achieved the highest 6-stage classification accuracy of 67.3% (95% CI: 65.5–69.2%), outperforming LM6_1 (58.8%, 95% CI: 55.4–62.2%) and LM6_2 (64.4%, 95% CI: 62.4–66.4%) (Table 2; Fig. 4). The quadratic weighted kappa for LS6 was 0.912 (95% CI: 0.902–0.922), compared to 0.889 (95% CI: 0.882–0.896) for LM6_1 and 0.900 (95% CI: 0.895–0.906) for LM6_2. In the tolerance-based analysis, LS6 showed a ± 1-stage accuracy of 94.3%, slightly higher than LM6_1 (92.9%) and LM6_2 (92.8%), while all three models reached 99.7% for ± 2-stage accuracy. The mean AUC was 0.966 for LS6, 0.957 for LM6_1, and 0.962 for LM6_2.

**Table 2:** Performance of 6-stage classification models.

Model	6-stage accuracy (95% CI)	± 1-stage accuracy	± 2-stage accuracy	Quadratic Kappa (95% CI)	Weighted F1-score	Weighted sensitivity	Weighted specificity	Weighted precision	Mean AUC
LS6	0.673 (0.655–0.692)	0.943	0.997	0.912 (0.902–0.922)	0.673	0.673	0.936	0.680	0.966
LM6_1	0.588 (0.554–0.622)	0.929	0.997	0.889 (0.882–0.896)	0.591	0.588	0.921	0.613	0.957
LM6_2	0.644 (0.624–0.664)	0.928	0.997	0.900 (0.895–0.906)	0.640	0.644	0.930	0.640	0.962

(LS6, end-to-end 6-stage model; LM6_1, landmark-guided partial fine-tuning model; LM6_2, landmark-guided deeper fine-tuning model; AUC, area under the receiver operating characteristic curve)

Data are presented as Mean (95% CI). CIs were calculated using a t-distribution (df = 4) based on 5-fold cross-validation.

**Fig. 5:**
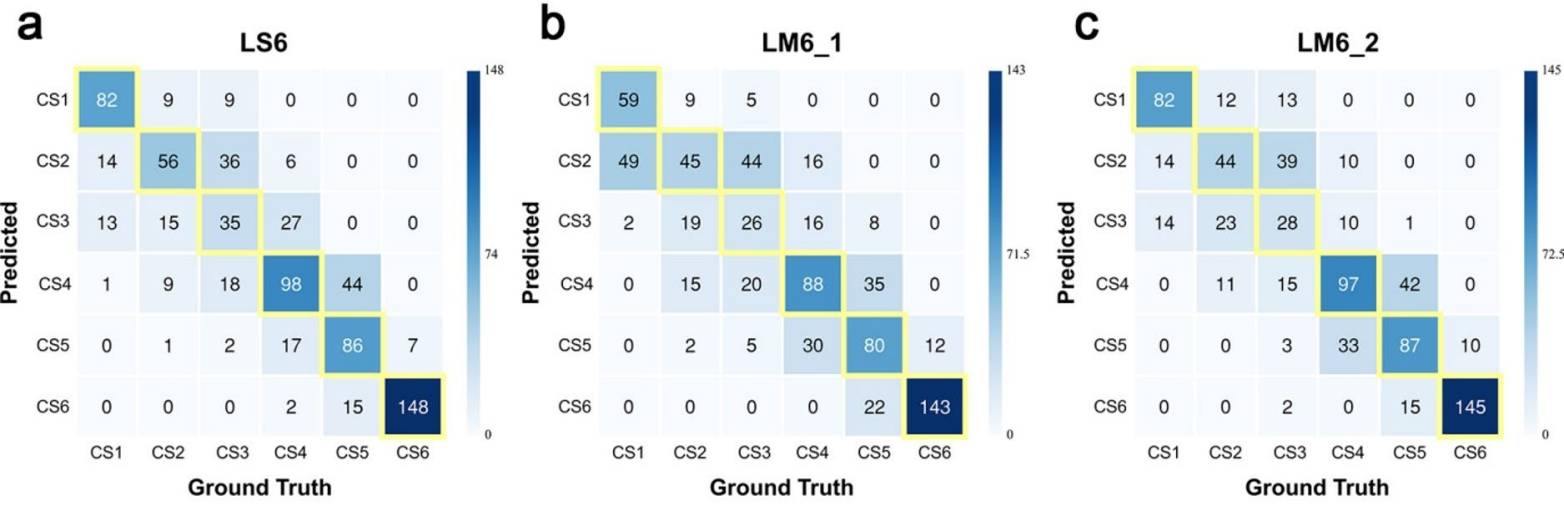
Confusion matrices for 6-stage classification. Confusion matrices for (**a**) LS6, (**b**) LM6_1, and (**c**) LM6_2. The yellow-outlined diagonal cells represent the true positive predictions for each stage (CS1–CS6). The concentration of values within these boxes demonstrates the classification accuracy of the model. LS6 shows the highest diagonal concentration, while LM6 models exhibit greater inter-stage dispersion. Most misclassifications occurred between adjacent stages across all models.

Stage-specific analysis revealed considerable performance variation across CVM stages (Table 3). CS3 showed the lowest accuracy across all three models (LS6: 35.0%, LM6_1: 26.0%, LM6_2: 28.0%). However, the confusion matrix (Fig. 5) showed that the majority of CS3 misclassifications were directed toward adjacent stages. In contrast, CS6 showed the highest accuracy, with all models exceeding 92%. The ± 1-stage accuracy of 94.3% demonstrates that errors across all stages were predominantly confined to adjacent maturation stages, with LS6 showing the strongest diagonal concentration in the confusion matrix (Fig. 5).

**Table 3:** Stage-specific accuracy of 6-stage classification models.

CVM Stage	LS6	LM6_1	LM6_2
CS1	0.745	0.536	0.745
CS2	0.622	0.500	0.489
CS3	0.350	0.260	0.280
CS4	0.653	0.587	0.647
CS5	0.593	0.552	0.600
CS6	0.955	0.923	0.935

(LS6, end-to-end 6-stage model; LM6_1, landmark-guided partial fine-tuning model; LM6_2, landmark-guided deeper fine-tuning model; CVM, cervical vertebral maturation; CS, cervical stage)

#### 3-stage classification: fine-to-coarse vs. direct learning (LS6_3 vs. LS3)

**Fig. 6:**
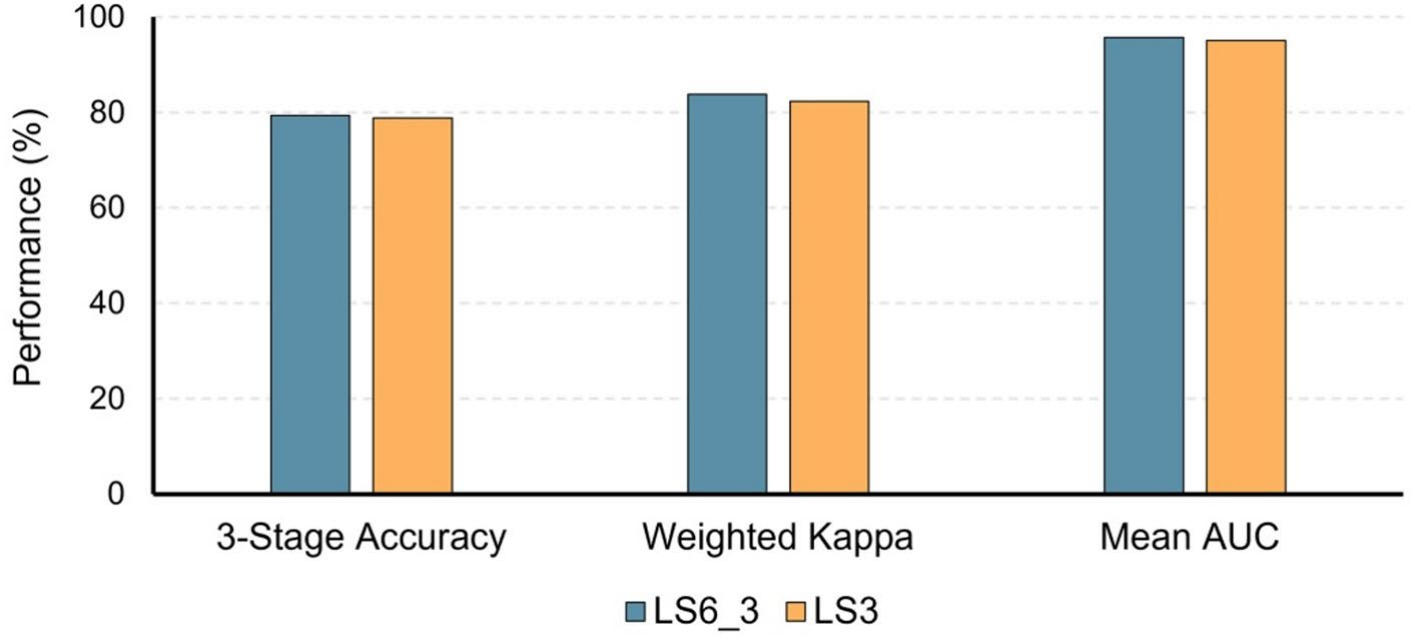
Performance comparison of 3-stage CVM classification models. Comparison of Accuracy, Quadratic Weighted Kappa, and Mean Area Under the Curve (AUC) between LS6_3 and LS3. LS6_3 represents the fine-to-coarse 3-stage classification derived from the 6-stage model (LS6), whereas LS3 is the model trained directly for 3-stage classification. LS6_3 achieved 79.3% accuracy, 0.838 weighted kappa, and 0.957 mean AUC, slightly outperforming LS3 across all metrics.

LS6_3 slightly outperformed LS3 across all major metrics (Table 4; Fig. 6). LS6_3 achieved an accuracy of 79.3% (95% CI: 76.6–82.1%) compared to 78.8% (95% CI: 76.7–80.9%) for LS3. The quadratic weighted kappa was 0.838 (95% CI: 0.814–0.862) for LS6_3 and 0.823 (95% CI: 0.805–0.841) for LS3. The mean AUC was 0.957 for LS6_3 and 0.951 for LS3. Stage-specific analysis of the 3-stage models showed that LS6_3 achieved notably higher accuracy than LS3 in the pubertal category (71.2% vs. 66.8%).

**Table 4:** Performance of 3-stage classification models.

Model	3-stage accuracy (95% CI)	Quadratic Kappa (95% CI)	Weighted F1-score	Weighted sensitivity	Weighted specificity	Weighted precision	Mean AUC
LS6_3	0.793 (0.766–0.821)	0.838 (0.814–0.862)	0.795	0.793	0.901	0.799	0.957
LS3	0.788 (0.767–0.809)	0.823 (0.805–0.841)	0.787	0.788	0.894	0.786	0.951

(LS6_3, fine-to-coarse 3-stage model; LS3, direct 3-stage model; AUC, area under the receiver operating characteristic curve)

Data are presented as Mean (95% CI). CIs were calculated using a t-distribution (df = 4) based on 5-fold cross-validation.

**Fig. 7:**
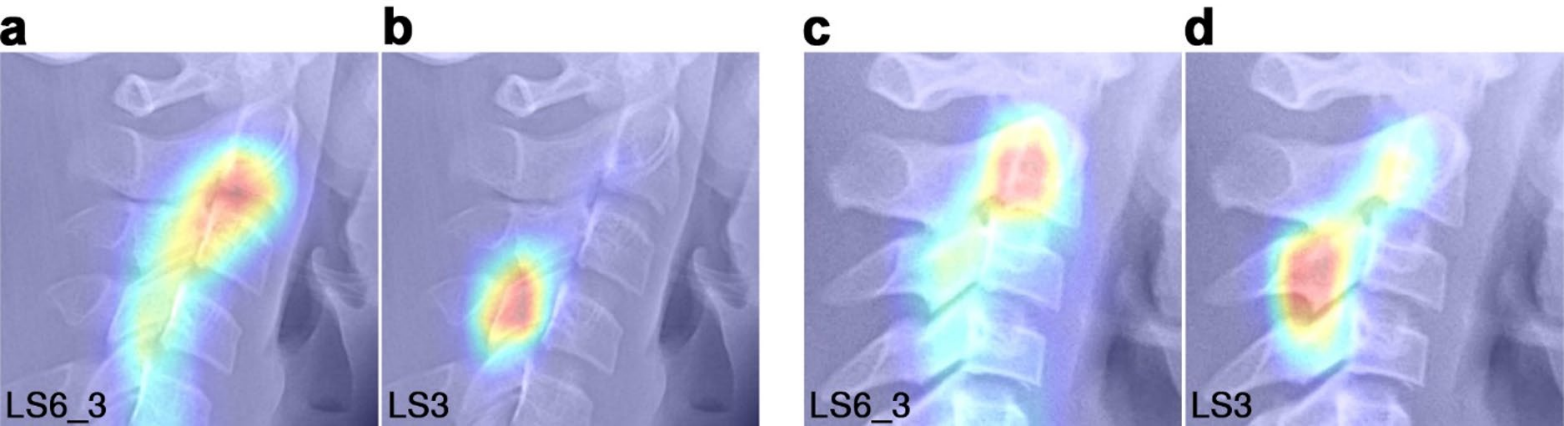
Grad-CAM visualization of model attention patterns. Grad-CAM heatmaps comparing anatomical focus between the two 3-stage models on representative cephalometric images. (**a**, **c**) LS6_3 model shows concentrated attention on clinically relevant features: inferior borders, anterior/posterior concavities, and vertebral body proportions. (**b**, **d**) LS3 model shows diffused activation including non-diagnostic areas.

Grad-CAM heatmaps revealed distinct differences in anatomical attention between the two 3-stage approaches (Fig. 7). LS6_3 showed concentrated activation on clinically relevant vertebral features, including the inferior cortical borders and the anterior/posterior concavities of the C2–C4 vertebral bodies. LS3 exhibited more diffuse activation patterns that extended to non-diagnostic peri-vertebral areas. This difference was consistently observed across multiple test cases.

## Discussion

Previous studies have attempted to automate CVM classification using deep learning, often incorporating anatomical landmarks as structural priors based on the premise that additional guidance would enhance model performance^9,13^. However, the actual contribution of landmark-based priors and the optimal level of classification granularity have not been systematically evaluated. This study addressed these gaps by comparing training strategies across two independent factors: structural priors and label granularity.

Contrary to expectations, the end-to-end model (LS6) outperformed the landmark-guided models (LM6_1, LM6_2) across all evaluation metrics. While deeper fine-tuning in LM6_2 partially improved performance over LM6_1, neither model matched LS6, suggesting that landmark-based priors did not confer a learning advantage for CVM staging. This result may be attributed to a task mismatch between landmark detection and CVM classification. Landmark detection emphasizes pixel-level localization of discrete anatomical points, whereas CVM staging requires the recognition of global morphological features such as vertebral silhouette, curvature, and proportional changes. When the source and target tasks are insufficiently aligned, transfer learning can lead to negative transfer, where pre-trained representations constrain rather than facilitate downstream learning^24,25^. This phenomenon has been documented in the transfer learning literature, where domain or task discrepancy between pre-training and fine-tuning stages results in degraded performance compared to training from scratch^26,27^. In our study, the landmark-pretrained backbone likely encoded a representational bias toward local geometric relationships, limiting the network’s capacity to capture the broader morphological context necessary for ordinal stage classification. The progressive improvement from LM6_1 to LM6_2 with deeper unfreezing further supports this interpretation, as it indicates that overwriting more of the landmark-biased features brought the model closer to—but did not reach—the performance of unconstrained end-to-end learning. It should be noted that this study tested one specific form of structural prior—sequential landmark-to-classification transfer learning. Alternative prior integration strategies, such as multi-task learning with joint landmark and stage optimization, or explicit shape-feature supervision, may yield different outcomes and warrant further investigation.

Another observation from the 6-stage analysis was the drop in accuracy at CS3 (35.0% for LS6), which was observed across all three models (Table 3). However, this observation likely reflects the intrinsic ambiguity of the CVM staging system rather than purely model limitations. Inter-clinician agreement for CVM staging is reported at 62–75%, with the greatest variability in the CS3–CS4 interval^28^, and a recent study found agreement as low as 42.82%^29^. This low agreement is largely attributable to the morphological ambiguity at CS3—the concavity of the inferior borders is developing but not yet fully established, making it difficult to distinguish from both CS2 and CS4. The confusion matrix showed that the majority of CS3 misclassifications were directed toward adjacent stages rather than clinically distant stages, which is consistent with the overall ± 1-stage accuracy of 94.3%. Correspondingly, although the overall 6-stage accuracy was 67.3%, the quadratic weighted kappa remained high (0.912), confirming that misclassifications were concentrated between neighboring stages rather than across clinically meaningful boundaries. This pattern is expected given that CVM stages represent discrete divisions of a continuous biological maturation process, and cases near stage boundaries are inherently ambiguous. The 3-stage classification framework effectively accommodates this inherent ambiguity by consolidating adjacent stages into broader clinically meaningful categories.

LS6_3 slightly outperformed LS3 in 3-stage classification, with accuracies of 79.3% and 78.8%, respectively. The confidence intervals for these values overlapped, indicating that the difference in classification accuracy was not statistically significant. However, the advantage of LS6_3 over LS3 was most pronounced in the pubertal category (71.2% vs. 66.8%), which is a clinically important stage for treatment timing decisions. This suggests that fine-grained 6-stage training may be particularly beneficial for distinguishing the diagnostically challenging pubertal growth period.

While the accuracy difference was marginal, Grad-CAM visualization (Fig. 7) revealed a difference in the quality of learned feature representations between the two 3-stage models. LS6_3 showed concentrated activation on diagnostically important structures—the inferior cortical borders, vertebral body concavities, and height-to-width proportions—whereas LS3 exhibited diffuse activation extending into non-diagnostic peri-vertebral areas. This pattern aligns with the concept of attention transfer^30^, where training on finer classification labels encourages the network to learn more discriminative features that are retained even when predictions are mapped to coarser categories. The attention pattern of LS6_3 aligns closely with the morphological criteria defined in the Baccetti CVM method, suggesting that 6-stage training guided the model toward the same anatomical features that clinicians use for staging. While the current Grad-CAM analysis is qualitative in nature, the consistent pattern observed across multiple test cases suggests that fine-grained training produces more anatomically grounded representations. This qualitative difference in attention patterns has practical implications beyond accuracy. A model that focuses on the same anatomical features used by clinicians may offer greater potential for clinical integration, as its predictions can be visually verified against established diagnostic criteria. This transparency is particularly relevant in orthodontic practice, where clinicians need to evaluate and, if necessary, override AI-generated recommendations before making treatment decisions.

Additional methodological refinements could further strengthen the clinical translation of this framework. Incorporating prediction calibration metrics such as Expected Calibration Error (ECE) would enable clinicians to assess the reliability of individual predictions, a prerequisite for clinical deployment. Additionally, direct comparison with clinician classifications on the same test set would provide a more rigorous benchmark for assessing clinical applicability^31^.

This study has several limitations. First, the fixed input resolution of 224 × 224 pixels, while standard for pretrained CNN architectures, may have limited the model’s capacity to capture subtle cortical concavity cues. Future studies should conduct a systematic ablation on input resolution and ROI configuration to determine whether higher-resolution inputs can improve stage-specific accuracy, particularly for transitional stages such as CS3. Second, the Grad-CAM analysis in this study was qualitative. Incorporating quantitative attention metrics, such as ROI overlap or attention concentration measures, would enable objective evaluation of model interpretability. Third, the single-institution nature of the dataset may restrict the generalizability of our findings across different imaging equipment and patient populations. Multi-center studies would be needed for comprehensive external validation. Although the dataset size is comparable to prior CVM AI studies, larger datasets may further improve model generalizability. Finally, this study focused exclusively on image-based assessment. In clinical practice, skeletal maturity assessment often integrates radiographic features with a broader clinical context, including chronological age, sex, and underlying systemic conditions. Further investigation of multimodal approaches that combine image data with structured clinical information to enhance decision-support accuracy is also warranted^32,33^.

In conclusion, this study compared training strategies for deep learning-based automated CVM staging across two independent factors. End-to-end learning outperformed landmark-guided transfer learning, indicating that task mismatch between landmark detection and CVM classification can lead to negative transfer. Fine-grained 6-stage training produced more anatomically focused attention patterns than direct 3-stage training, despite comparable classification accuracy. These findings provide preliminary evidence for optimizing AI training protocols in skeletal maturity assessment, though multi-center validation is needed before clinical implementation. More broadly, they highlight that model training strategy can influence not only predictive accuracy but also the anatomical plausibility of learned representations, which may be important for clinician trust in AI-assisted diagnosis.

## Data Availability

Data supporting the findings of the current study are available from the corresponding author upon reasonable request. The code and model architecture details will also be made available upon reasonable request.
